# Endless Forms Most Viral

**DOI:** 10.1371/journal.pgen.1001210

**Published:** 2010-11-18

**Authors:** Welkin E. Johnson

**Affiliations:** New England Primate Research Center, Department of Microbiology and Molecular Genetics, Harvard Medical School, Southborough, Massachusetts, United States of America; Fred Hutchinson Cancer Research Center, United States of America

Perhaps more than any other biological discipline, the study of animal viruses is confined to the present. Virions are simply not the stuff of which robust fossils are made. Phylogenetic analysis can help by revealing deep relationships between extant viral lineages, yet such reconstructions lack detail (telling us nothing about transitional or extinct viral forms, the movement of viruses between species, or the timing of major events in viral evolution), and molecular clock estimates are notoriously imprecise when applied to viruses [1]. Until recently, ancient endogenous retroviruses (ERVs) were the closest thing to a fossil record available to scientists with a proclivity for combining virology and natural history. Happily, a trio of recent studies appearing in *PLoS Genetics* [2], *PLoS Biology* [3], and *PLoS Pathogens* [4] reveal an unexpected wealth of non-retroviral virus sequences embedded in the genome sequence databases, a virtual equivalent of the Burgess Shale, ripe for excavation by eager paleovirologists.

Retroviral infection occasionally results in the deposition of a provirus in a host's germline DNA. While germline integration of a provirus may be an exceedingly rare event, across the great expanse of evolutionary time millions of ERV loci have accumulated in animal genomes. Because retroviruses replicate through an integrated DNA intermediate, it is not difficult to imagine how ERVs are generated. For other animal viruses, which do not normally integrate their genomes into host DNA, the formation of germline insertions should be far less likely. Nonetheless, reports of non-retroviral specimens being unearthed from the genomes of animal species are on the rise. Notable examples include functional expression of nudivirus-related structural genes in the genomes of parasitic wasps [5]; Ebolavirus-like sequences, related to modern filoviruses, present in multiple mammalian genomes [6]; and sequences resembling the Bornavirus nucleoprotein gene (N) in the genomes of various mammals including primates, rodents, and elephants [7]. Even some herpesviruses have a propensity for occasional germline insertion and thus, the potential for vertical inheritance [8]. Now, Belyi et al. [4] and Katzourakis and Gifford [2], have unearthed diverse collections of non-retroviral sequences buried in whole genome sequence data from an impressive array of host organisms, including mammals, marsupials, birds, rodents, and insects, using modern viral sequences as bioinformatic probes. A third study from Gilbert and Feschotte specifically reevaluates the macroevolution of hepadnaviruses based on the sequence and distribution of hepadnavirus-like fossils in the genomes of passerine birds [3]. To cope with this newfound abundance, the authors of one of the studies suggest the acronym EVE (for endogenous viral element) as a general term to encompass all virus-derived genomic loci [2].

Two of the studies also took a closer look at a previously described class of EVEs, called EBLNs (for endogenous Bornavirus-like N genes) [2], [4], [7]. While most EVEs were either defective at the time of insertion or rendered functionless by the accumulation of random mutations over the course of millions of years, EBLNs are striking in retaining largely intact protein-coding sequences. In fact, in silico simulations of EBLN evolution estimate that these elements should have accumulated ∼10–20 stop codons since the time of genome insertion. That the EBLN coding sequences appear relatively unscathed suggests that these particular elements provide (or at times provided) a selectively advantageous function, subjecting them to purifying selection. The possibility is not without precedent: for example, at least one human ERV has evolved to provide a cellular function [9], and there are several examples of ERVs that have been subverted by host evolution to serve as inhibitors of retroviral infection [10]–[14].

As a group, viruses are polyphyletic, as evidenced by the variety of unique genome types and distinctive replication strategies they collectively employ. There are double-stranded DNA viruses and single-stranded DNA viruses, double-stranded and single-stranded RNA viruses, and viruses with segmented genomes; among those with single-stranded RNA, there are those with positive polarity (the genome resembles an mRNA) and those with negative sense genomes. Each genome type represents a different starting point for takeover of the host cell, and each requires a different strategy for achieving this fundamental task. For example, replication of some viruses is confined entirely to the cytoplasm, whereas others involve synthesis of DNA or RNA in the nucleus. While the fossil record is still dominated by retroviral sequences, the inventory of known EVE loci now appears to include representatives of all the basic replication strategies exemplified by modern viruses. Non-retroviral EVEs are typically subgenomic, derived from just one or a few viral genes instead of entire viral genomes. Insertion site duplications bracketing some EVEs suggest that retrotransposition *in trans*, by retrotransposons or possibly retroviruses, may be a predominant mechanism of EVE formation. In fact, for RNA viruses that replicate in the cytoplasm (e.g., filoviruses and rhabdoviruses), retrotransposition is the most plausible mechanism for EVE formation. In such cases, it will be interesting to determine whether the more abundant EVE sequences share some common feature(s) conferring a propensity for retrotransposition. In contrast, hepadnavirus “fossils” lack the hallmarks of retrotransposition (such as flanking insertion-site duplications and poly-A tails), and may instead have resulted from non-homologous end joining and insertion of viral DNA directly into the host genome [3].

When incorporated into phylogenetic trees, many EVEs group as sister taxa to their modern counterparts. Thus, they are not evolutionary intermediates on the path to extant viruses, but rather extinct lineages sharing a common ancestor with modern viruses. From this, one can infer that most of the distinctive replication strategies employed by modern viruses probably originated hundreds of millions of years ago. While virologists intuitively understand this (given the widespread distribution of viruses among living organisms), EVEs constitute direct, physical evidence that modern viral lineages have very ancient roots (Table 1). That modern viruses and ancient EVE sequences are still recognizably related is astonishing, given that they are separated by millions of years of exogenous viral evolution.

**Table 1:** Estimated Minimum Age of Select EVEs.

Modern Viruses	Est. Minimum Age (Based on Related EVE)	Genome	Reference
Lentiviruses	≥2–4 MYA	Single-strand RNA (+); reverse transcribing	[15], [16], [17]
Spumaviruses	100 MY	Single-strand RNA (+); reverse transcribing	[18]
Bornaviruses	93 MY	Single-strand RNA (-)	[2], [4], [7]
Filoviruses	30 MY12–24 MY	Single-strand RNA (-)	[2], [4], [6]
Circoviruses	68 MY	Single-strand DNA	[2]
Hepadnaviruses	19 MY	Double-strand DNA, gapped circular	[2], [3]

MY, millions of years.

**Figure 1:**
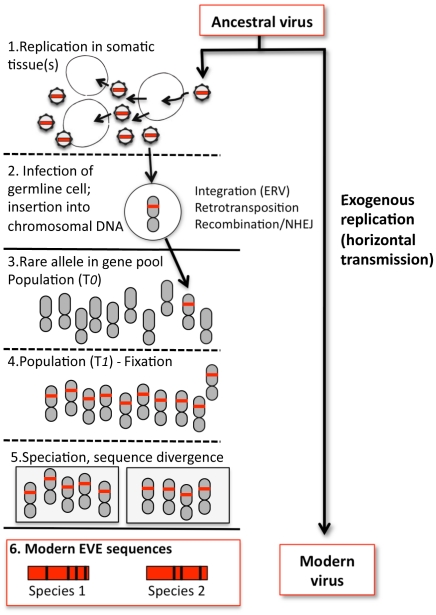
Formation of a hypothetical EVE and relationship to modern viruses. 1. An ancestral virus spreads in a host population, infecting and replicating in somatic tissue(s) of infected individuals. 2. Occasionally, a virion may encounter a germline cell (or any cell in the developmental pathway leading to germline tissue); in some cases viral sequence is inserted into chromosomal DNA. For retroviruses, integration is an essential step in viral replication; for other viruses, insertion is a rare by-product of replication and must be mediated by other mechanisms, such as retrotransposition *in trans* or recombination. In addition, any given virus may not efficiently infect or replicate well in such cells, reducing the probability of insertion. Likewise, infections or insertions that are deleterious to the cell or tissue will reduce the probability of vertical transmission. 3. If gametes bearing the insertion are formed and the chromosome bearing the viral sequence is inherited, the insertion initially exists as a rare allele (the majority of individuals lack the insertion) and the fate of the newborn EVE is similar to any other chromosomal mutation, subject to loss or fixation by random genetic drift (if the insertion has phenotypic consequences, natural selection may also play a role). 4. More often than not, EVEs are probably lost by chance. On rare occasions, an insertion may drift towards higher frequency. Early on, speciation events and incomplete lineage sorting can lead to fixation in some lineages but not others, and chance extinction of populations with the insertion can still lead to loss (only fixation is shown). 5. In descendant species that share the insertion, the orthologous EVE loci will evolve independently. 6. The genetic distance between orthologous EVEs in the genomes of modern species reflects the time passed since the last common ancestor of these species, and provides a lower bound estimate of the time since insertion. Divergence between EVE sequences and the sequences of their modern viral relatives is the combined result of EVE evolution (as part of the nuclear genome) and exogenous viral evolution, the rates of which can differ by several orders of magnitude.

The catalog of EVEs is impressive for what it contains, but even more so for what it does not. Why? Because the known EVEs probably represent a minor and highly skewed sampling of viral prehistory. Minor, because the odds that infection of an individual organism will result in fixation of an EVE are exceedingly small (Figure 1). Skewed, because some viruses may be more prone to germline insertion than others (that retroviral insertions greatly outnumber other EVEs is a particularly striking example of a virus-dependent bias). Thus, as impressive in scope and variety as the EVEs are, they may represent but a drop in the ocean of all the viruses that have buffeted host organisms across the ages.

The current EVE record may have other limitations. Just how far back does the EVE fossil record extend? Erosion due to the steady accumulation of mutations must impose an upper limit on how far back the viral fossil record can be deciphered, and theoretical predictions of that limit would be useful. Even in the absence of sequence degradation, some EVEs may be easier to detect than others. For example, the studies described here relied on known viral sequences as queries: if our genomes also harbor ancient viral sequences for which there is no modern counterpart, how would we recognize them for what they once were?
